# Tumor Whole-Genome Sequencing for Prediction of Venous Thromboembolism in Patients With Metastasized Solid Cancer

**DOI:** 10.1161/CIRCGEN.124.005182

**Published:** 2026-04-10

**Authors:** Frits I. Mulder, Noori A.M. Guman, Job van Riet, Roos J. van Geffen, Thijs F. van Haaps, Vahram Hovsepjan, Hanneke W.M. van Laarhoven, Geert A. Cirkel, Filip Y.F.L. de Vos, Hans M. Westgeest, Laurens V. Beerepoot, Judith de Vos-Geelen, Mariette Labots, Paul Hamberg, Annelie J.E. Vulink, Maartje Los, Aeilko H. Zwinderman, Debbie G.J. Robbrecht, Maja J.A. de Jonge, Harry R. Büller, Pieter W. Kamphuisen, Bart Ferwerda, Neeltje Steeghs, Nick van Es

**Affiliations:** 1 Department of Vascular Medicine, Amsterdam UMC Location University of Amsterdam, the Netherlands (F.I.M., N.A.M.G., R.J.v.G., T.F.v.H., V.H., H.R.B., P.W.K., N.v.E.).; 2 Amsterdam Cardiovascular Sciences, Pulmonary Hypertension and Thrombosis, the Netherlands (F.I.M., N.A.M.G., R.J.G., T.F.H., V.H., H.R.B., P.W.K., N.v.E.).; 3 Department of Internal Medicine, Tergooi MC, Hilversum, the Netherlands (F.I.M., N.A.M.G., P.W.K., N.v.E.).; 4 Division of AI in Oncology, German Cancer Research Ctr DKFZ, Heidelberg, Germany (J.v.R.).; 5 Department of Medical Oncology, Amsterdam UMC Location Vrije Universiteit Amsterdam, the Netherlands (F.I.M., H.W.M.v.L., M. Labots).; 6 Department of Internal Medicine, Meander Medical Ctr, Amersfoort, the Netherlands (G.A.C.).; 7 Department of Medical Oncology, University Medical Ctr Utrecht, the Netherlands (F.Y.F.L.d.V.).; 8 Department of Internal Medicine, Amphia Hospital, Breda, the Netherlands (H.M.W.).; 9 Department of Internal Medicine and Medical Oncology, Elisabeth-Tweesteden Hospital, Tilburg, the Netherlands (L.V.B.).; 10 Department of Medical Oncology, GROW, Maastricht University Medical Centre, the Netherlands (J.d.V.-G.).; 11 Cancer Centre Amsterdam, Imaging & Biomarkers, the Netherlands (H.W.M.L., M. Labots).; 12 Department of Internal Medicine, Franciscus Gasthuis & Vlietland, Rotterdam, the Netherlands (P.H.).; 13 Department of Internal Medicine, Reinier de Graaf Hospital, Delft, the Netherlands (A.J.E.V.).; 14 Department of Internal Medicine, St Antonius Hospital, the Netherlands (M. Los).; 15 Department of Clinical Epidemiology, Biostatistics & Bioinformatics, Amsterdam University Medical Centre, the Netherlands (A.H.Z., B.F.).; 16 Department of Medical Oncology, Erasmus Medical Centre, Rotterdam, the Netherlands (D.G.J.R., M.J.A.d.J.).; 17 Department of Medical Oncology, Netherlands Cancer Institute, Amsterdam (N.S.).

**Keywords:** anticoagulants, germ cells, humans, mutation, neoplasms

## Abstract

**BACKGROUND:**

International guidelines suggest prophylactic anticoagulation for patients with cancer at high risk of venous thromboembolism (VTE). Here, we evaluated whether tumor whole-genome sequencing data may improve the selection of high-risk patients.

**METHODS:**

In a pan-cancer cohort of 3087 patients, associations of candidate clinical predictors, the germline extended 297-single nucleotide polymorphism polygenic risk score, tumor mutational characteristics, and somatic tumor mutations with VTE were estimated by calculating hazard ratios (HRs).

**RESULTS:**

During 12-month follow-up, 237 (7.7%) developed VTE. The germline extended 297-single nucleotide polymorphism score was associated with VTE (HR per point increase, 2.11 [95% CI, 1.45–3.06]), as well as the total number of somatic structural variants (HR per 1000 increase, 1.21 [95% CI, 1.07–1.37]) and 129 somatic mutations (unadjusted *P*<0.05), including *POLR2E* (HR, 3.34 [95% CI, 1.68–6.97]), *PALM* (HR, 3.73 [95% CI, 1.74–7.99]), *TBX22* (T-box Transcription Factor 22; HR, 2.47 [95% CI, 1.40–4.35]), and *ELANE* (HR, 3.22 [95% CI, 1.51–6.87]). To further explore the potential of tumor whole-genome sequencing, prediction models were constructed including selected clinical predictors only (model 1), clinical predictors and germline variants (model 2), and the combination of clinical predictors, germline variants, and 14 top discriminating somatic mutations (model 3). The optimism corrected concordance index was 0.66 (95% CI, 0.62–0.69) for model 1, 0.67 (95% CI, 0.62–0.72) for model 2, and 0.77 (95% CI, 0.72–0.81) for model 3. This was significantly higher than that of the currently endorsed clinical Khorana VTE risk score (concordance index, 0.55 [95% CI, 0.51–0.59]; *P*<0.005).

**CONCLUSIONS:**

These data indicate that tumor whole-genome sequencing may improve VTE prediction by clinical risk scores. Validation studies to confirm these findings are needed.

The association between cancer and venous thromboembolism (VTE)—comprising pulmonary embolism and deep vein thrombosis (DVT)—has been documented since the early 19th century.^1^ The risk is about 20-fold higher in patients with cancer receiving systemic therapy than in the general population, but the incidence rate varies substantially across cancer types.^2^ Intriguingly, this variation in VTE risk is already present just before cancer is diagnosed. This observation suggests that intrinsic tumor features drive the hypercoagulable state independent of treatment-related factors such as systemic cancer therapy, surgery, and hospitalization.^2,3^

Cancer-associated VTE leads to morbidity, mortality, decreased quality of life, and increased health care costs.^4–8^ Therefore, international guidelines recommend pharmacological thromboprophylaxis for 6 months for ambulatory cancer patients at high risk of VTE, who can be identified using the Khorana VTE risk score.^9–11^ This score uses 5 readily available clinical variables (cancer type, hemoglobin level, leukocyte count, platelet count, and body mass index) to stratify patients into low- and high-risk groups. However, its discrimination is modest, with reported area under the receiver operating characteristic curves ranging from 0.55 to 0.65, which calls for efforts to improve prediction.^12–14^

Besides clinical risk factors, genetic variations in germline DNA (called germline variants) and tumor tissue DNA (called somatic mutations) may contribute to the risk of VTE. In the general population, factor V Leiden (rs6025),^15^ prothrombin G20210A (rs1799963),^16^ and various germline polygenic risk scores are strongly associated with VTE, but data on their predictive performance in cancer patients are very limited.^17^ The association between whole-genome somatic mutations, and VTE has not been evaluated. Certain tumor driver mutations, such as *KRAS*, *STK11*, and *MET*, were previously linked to VTE risk,^18–21^ but they form only a small subset of the total tumor mutational landscape (<0.02%).^22^

Therefore, the aim of this study was to evaluate the added value of prothrombotic germline variants and whole-genome somatic mutations, conditional on clinical predictors, for prediction of VTE in cancer patients. We assessed whether these clinical and genetic predictors could be combined in an exploratory prediction model.

## Methods

**Figure 1.**
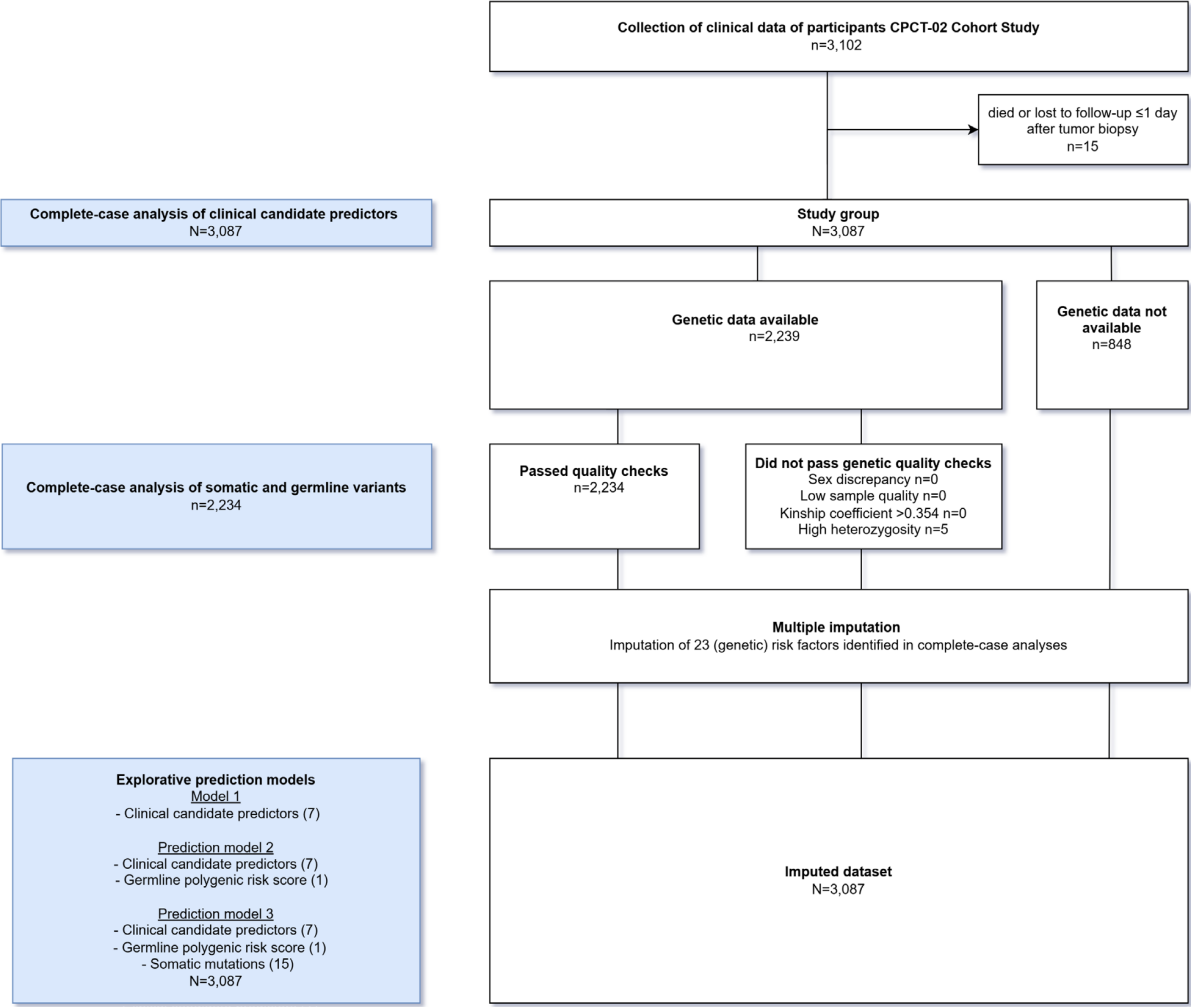
Study overview.

We used data from the CPCT-02 study (Center for Personalized Cancer Treatment-02), in which whole-genome germline and tumor data were collected from patients with metastatic or locally advanced solid cancer if they were eligible for systemic cancer therapy.^23^ Full methodology on the CPCT-02 cohort, and methods outlining design, ethical approval, outcomes, imputation, and statistical analysis of the present study are provided in the Supplemental Methods. Data are available upon request at the Hartwig Medical Foundation. Figure 1 shows the flow chart of the study. For the present analysis, additional data on patient characteristics at the time of inclusion in the CPCT-02 study and on venous or arterial thromboembolic outcomes were retrospectively collected from electronic patient records in 12 cancer centers in the Netherlands. VTE events were adjudicated by 2 authors by carefully assessing radiology reports and information from patient records.

Patients were followed from the day of biopsy until 12 months, death, or loss to follow-up. The primary outcome was VTE, defined as the composite of symptomatic and incidental pulmonary embolism, proximal or distal DVT of the lower or upper extremities, splanchnic vein thrombosis, and cerebral vein thrombosis. The secondary outcome was a stricter definition of VTE, defined as pulmonary embolism or lower-extremity DVT.

The associations between selected clinical candidate predictors, germline variants, and somatic tumor mutations with VTE were evaluated in complete-case analyses using Cox proportional hazards regression models. Clinical candidate predictors were selected based on existing literature (see Supplemental Text 1 for details on methods). Cancer type was classified as very high risk for VTE (glioblastoma, gastric, pancreatic, or hepatobiliary cancer), high risk for VTE (malignant brain tumors other than glioblastoma, multiple myeloma, lymphoma, and lung, testicular, renal, esophageal, colorectal, bladder, or gynaecological cancer), or low risk (all other cancer types; Table S2).

The germline candidate predictors included a polygenic risk score (extended 297-single nucleotide polymorphism [SNP] score) and genetically determined blood group type non-0. The extended 297-SNP score consists of a 297-SNP score plus factor V Leiden and the prothrombin gene mutation.^17^ These predictors were chosen based on a previous analysis among 36 150 cancer patients enrolled in the UK Biobank study.^24^ The tumor mutational characteristics (eg, total number of structural variants) and all somatic mutations with a mutational prevalence of >1.5% (n>34) were explored for their association with VTE. All analyses on tumor mutational characteristics and somatic mutations were adjusted for cancer type using the aforementioned VTE risk cancer groups. We considered clinical predictors and somatic mutations associated with VTE based on an unadjusted *P*<0.05 for further prediction modeling. Missing data on these somatic mutations, as well as germline data that were identified as statistically significant candidate predictors in the complete-case analyses, were subsequently imputed in 27 data sets. Three exploratory multivariable Cox prediction models were constructed including clinical predictors only (model 1), clinical predictors and germline variants (model 2), and clinical predictors, germline variants, and somatic mutations (model 3). In model 3, only somatic mutations with a prevalence of >2.5% (n>56) were included to reduce the risk of chance findings.

Discrimination of the models was assessed and compared with the Khorana score by calculating the concordance index (C-index) with 95% CIs using the DeLong method. The C-index was adjusted for optimism by repeating the modeling steps in 250 bootstrap samples in the 27 imputed data sets. Calibration of model 3 was evaluated by plotting the estimated probability of VTE (in deciles) versus the observed 12-month cumulative incidence of VTE, in which death not related to VTE was considered a competing risk.^25^

In addition, the cumulative incidence of VTE was calculated in high- and low-risk groups based on the Khorana score (≥2 versus <2 points^26,27^) as well as based on model 3. For model 3, a positivity threshold was pragmatically chosen so that the proportion of high-risk patients matched that of the Khorana score at its positivity threshold of ≥2 points.^28^ Fine and Gray models were used to calculate subdistribution HRs (SHRs) between the high- and low-risk groups.

All analyses were performed using R software (R Foundation for Statistical Computing, Vienna, Austria; https://www.R-project.org/), in particular using the packages *psfmi*, *mice*, and *rms*. A *P*<0.05 was considered statistically significant. This article adheres to the Transparent Reporting of a Multivariable Prediction Model for Individual Prognosis or Diagnosis statement (see Table S1 for checklist).^29^

This study complies with the Transparency and Openness Promotion guidelines. Data, analytic methods, and materials are available upon reasonable request, subject to approval by the Hartwig Medical Foundation and the relevant institutional review boards. The CPCT-02 study was approved by the institutional review boards of all participating centers, and all patients provided written informed consent for participation in the CPCT-02 study. The current analysis was additionally approved by the institutional review boards of all participating centers.

## Results

### Study Group and Outcome

Data were obtained from 3102 cancer patients of whom 15 (0.5%) were excluded because they died or were lost to follow-up within 1 day after tumor biopsy. Baseline characteristics of the remaining 3087 patients are shown in Table 1. The median age was 64 years (interquartile range, 55–70), and 1500 (48.6%) patients were women. The most frequent cancer types were breast cancer (17.0%), colorectal cancer (11.9%), melanoma (11.7%), and lung cancer (11.5%). The median follow-up time was 281 days (interquartile range, 105–365). During the 12-month observation period, 1388 (44.9%) patients died, 243 (7.8%) were lost to follow-up, and 237 (7.7%) were diagnosed with VTE. Of the 237 patients with VTE, 125 (52.7%) had pulmonary embolism, 59 (24.9%) had lower-extremity DVT, 21 (8.9%) had upper-extremity DVT, 26 (11.0%) had splanchnic vein thrombosis, and 6 (2.5%) had other types of VTE. The median time between biopsy and VTE was 96 days (interquartile range, 48–170). Germline and whole-genome tumor mutational predictors were evaluated in 2234 of 3087 (72.4%) patients with available quality-checked genetic data (see Figure 1 for flowchart). No violations of the proportional hazards assumption were observed based on Schoenfeld residuals.

**Table 1.**
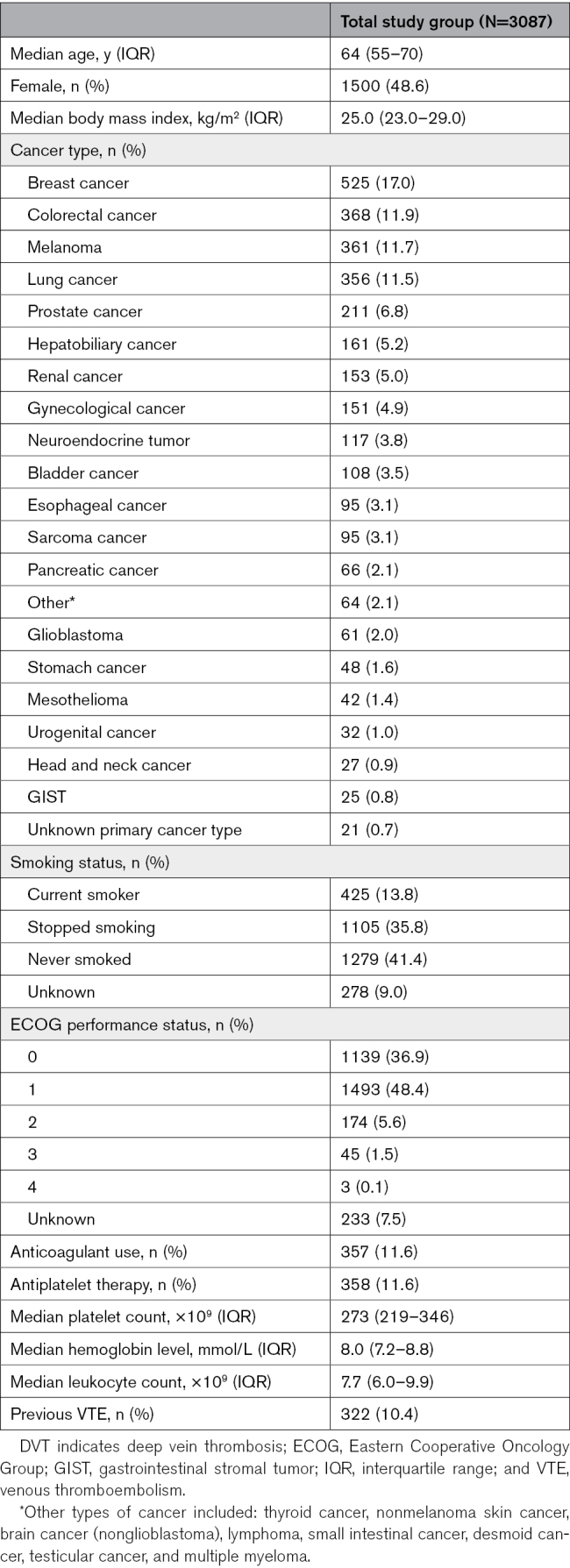
Baseline Characteristics of Study Group

### Clinical Predictors for Venous Thromboembolism

Among the 3087 cancer patients with clinical data, cancer types classified as high risk (HR, 2.23 [95% CI, 1.67–2.98]) and very high risk (HR, 3.12 [95% CI, 2.14–4.55]) were significantly associated with VTE as compared with other cancer types. Other baseline clinical predictors associated with VTE in univariable analyses (unadjusted *P*<0.05) were log-transformed leukocyte count (HR per log increase, 2.17 [95% CI, 1.58–2.99]), treatment with platinum-based chemotherapy (HR, 2.01 [95% CI, 1.54–2.64]), Eastern Cooperative Oncology Group performance status ≥2 (HR, 2.16 [95% CI, 1.40–3.33]), prior VTE (HR, 1.63 [95% CI, 1.14–3.34]), log2-transformed platelet count (HR per log2 increase, 1.33 [95% CI, 1.04–1.70]), and squared hemoglobin count (HR per squared hemoglobin count increase, 0.992 [95% CI, 0.985–0.9998]; Table 2).

**Table 2.**
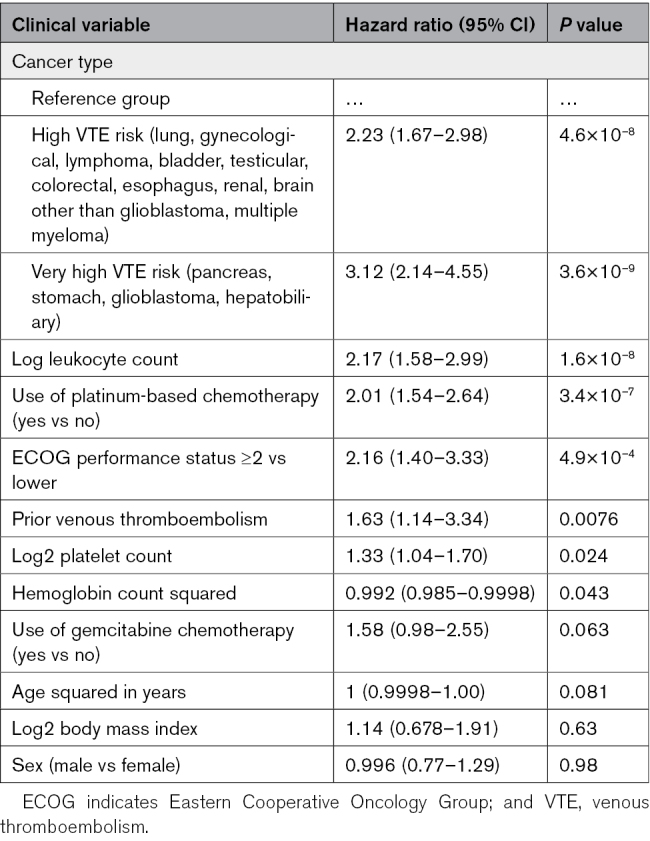
Univariable Association Between Clinical Predictors and Venous Thromboembolism During 12 Months Follow-Up

### Germline Predictors for Venous Thromboembolism

Among the 2234 cancer patients with available quality-checked genetic data, blood group non-O (n=1230) was not associated with VTE (HR, 1.01 [95% CI, 0.75–1.36]). The C-index for the continuous extended 297-SNP score was 0.57 (95% CI, 0.51–0.61), and the HR was 2.11 (95% CI, 1.45–3.06) per point increase.

### Tumor Mutational Predictors for Venous Thromboembolism

Of the 7501 somatic gene mutations in tumor tissue with a prevalence >1.5%, 129 were associated with VTE during 12-month follow-up following tumor biopsy based on an unadjusted *P*<0.05 (Table S3). The 10 genes with the strongest association with VTE are shown in Table 3, and included *POLR2E* (RNA Polymerase II Subunit E; HR, 3.34 [95% CI, 1.68–6.97]), *PALM* (Paralemmin; HR, 3.73 [95% CI, 1.74–7.99]), *TBX22* (T-box Transcription Factor 22; HR, 2.47 [95% CI, 1.40–4.35]), and *ELANE* (Elastase, Neutrophil Expressed; HR, 3.22 [95% CI, 1.51–6.87]). Of the evaluated tumor mutational characteristics, the total number of structural variants (HR per 1000 increase, 1.21 [95% CI, 1.07–1.37]) and whole-genome duplication (HR, 1.10 [95% CI, 1.01–1.21]) were associated with VTE (Table 3).

**Table 3.**
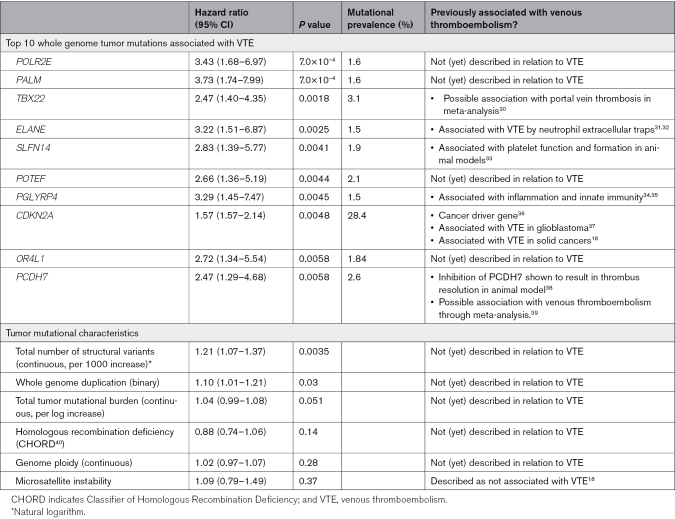
Association Between Tumor Genetics and Venous Thromboembolism During 12 Months Follow-Up

### Exploratory Prediction Models

The imputed data set comprising all 3087 patients was used to develop 3 exploratory prediction models. Full model 1 included the following 7 clinical candidate predictors based on their association with VTE in the complete-case analysis: cancer type risk group, leukocyte count, platinum-based chemotherapy, Eastern Cooperative Oncology Group performance status, prior VTE, platelet count, and hemoglobin level. After stepwise backward selection, 5 variables were retained in final model 1 (cancer type risk group, leukocyte count, platinum-based chemotherapy, Eastern Cooperative Oncology Group performance status, and prior VTE), which had an optimism-adjusted C-index of 0.66 (95% CI, 0.62–0.69).

In full model 2, the same 7 clinical candidate predictors were included together with the extended 297-SNP polygenic risk score. All items were retained after stepwise backward selection, except for hemoglobin level and platelet count. Final model 2 had an optimism-adjusted C-index of 0.67 (95% CI, 0.62–0.72).

**Figure 2.**
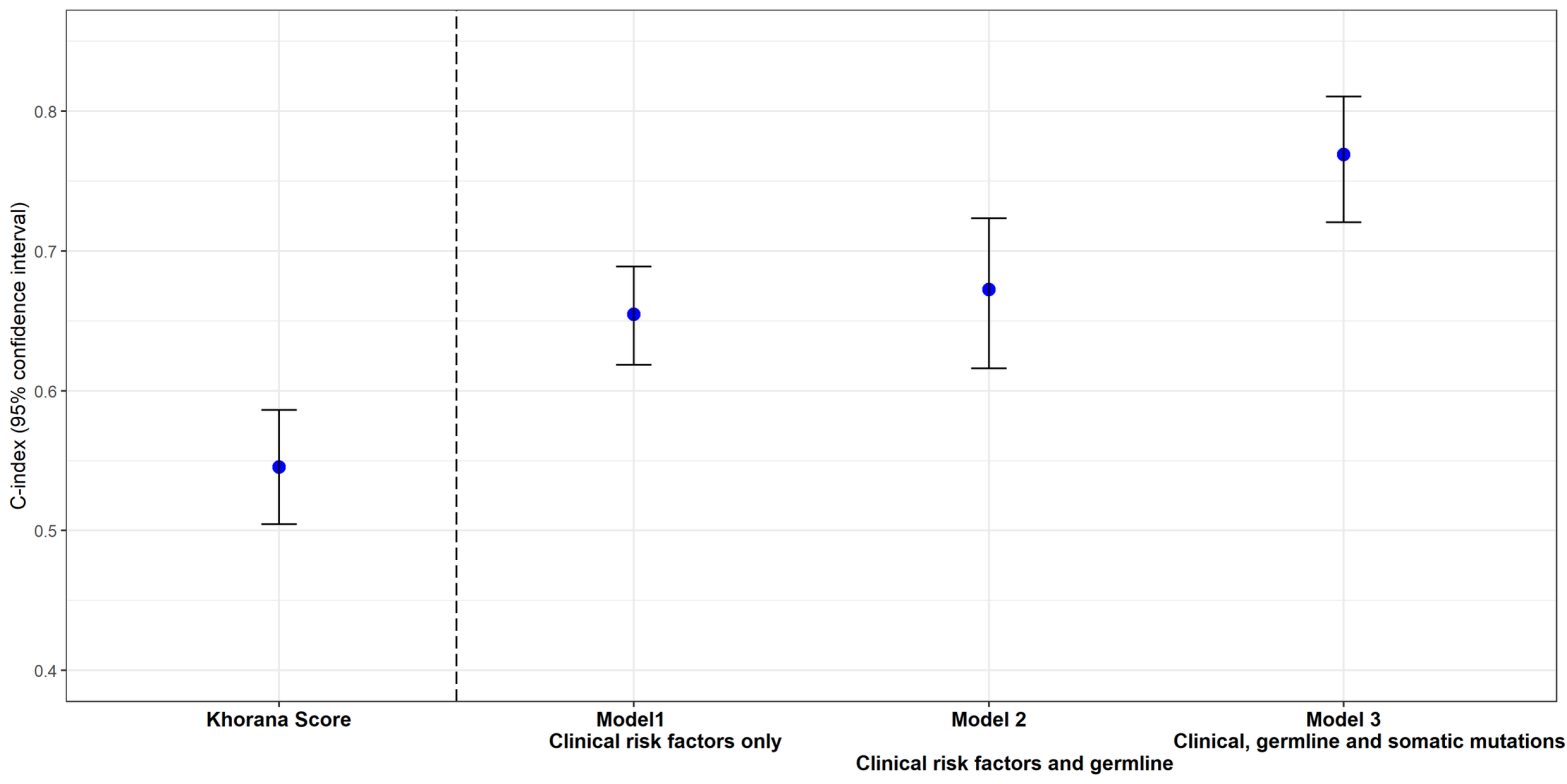
Optimism-adjusted C-index of the Khorana score and of the 3 exploratory prediction models.

Full model 3 comprised the 7 clinical candidate predictors, the extended 297-SNP polygenic risk score, the top 14 tumor whole-genome somatic mutations with a prevalence ≥2.5%, and the total number of structural variants. The following items were retained in final model 3: cancer type VTE risk group, leukocyte count, Eastern Cooperative Oncology Group performance status score ≥2, platinum-based chemotherapy, prior VTE, extended 297-SNP polygenic risk score, total number of structural variants, and the somatic mutations *TBX22, CDKN2A* (Cyclin Dependent Kinase Inhibitor 2A), *PCDH7* (Protocadherin 7), *COL22A1* (Collagen Type XXII Alpha 1 Chain), *ADAMTS20* (A Disintegrin and Metalloproteinase with Thrombospondin Motifs 20), *CSMD3* (CUB and Sushi Multiple Domains 3), *PLEC* (Plectin), *FRMPD4* (FERM and PDZ Domain Containing 4), *WDR64* (WD Repeat Domain 64), *BRINP2* (BMP/retinoic acid-inducible neural-specific protein 2), *PCDHGA4* (Protocadherin Gamma Subfamily A, 4), *XKR3* (XK Related 3), *ASMT* (Acetylserotonin O-methyltransferase), and *KDM6A* (Lysine Demethylase 6A). The optimism-adjusted C-index of this final model was 0.77 (95% CI, 0.72–0.81) (Figure 2). The calibration plot of model 3 is shown in Figure S1. The model overestimated VTE probability in the lower range (<10%) but was reasonably well calibrated in the higher range of estimated probabilities. Details of the full and final models 1, 2, and 3 are provided in Table S4.

The C-index of the Khorana VTE score, the current clinical benchmark, was 0.55 (95% CI, 0.51–0.59), which was significantly lower than the optimism-adjusted C-index of model 3 (*P*<0.005). In the imputed data sets, 24% of patients had a Khorana score ≥2 points (high-risk group) and 76% had a score <2 points (low-risk group). The 12-month VTE cumulative incidence was 9.5% (95% CI, 7.4–11.9) in the Khorana score high-risk group compared with 7.1% (95% CI, 6.1–8.2) in the Khorana score low-risk group (subdistribution hazard ratio, 1.37 [95% CI, 1.05–1.70]).

**Figure 3.**
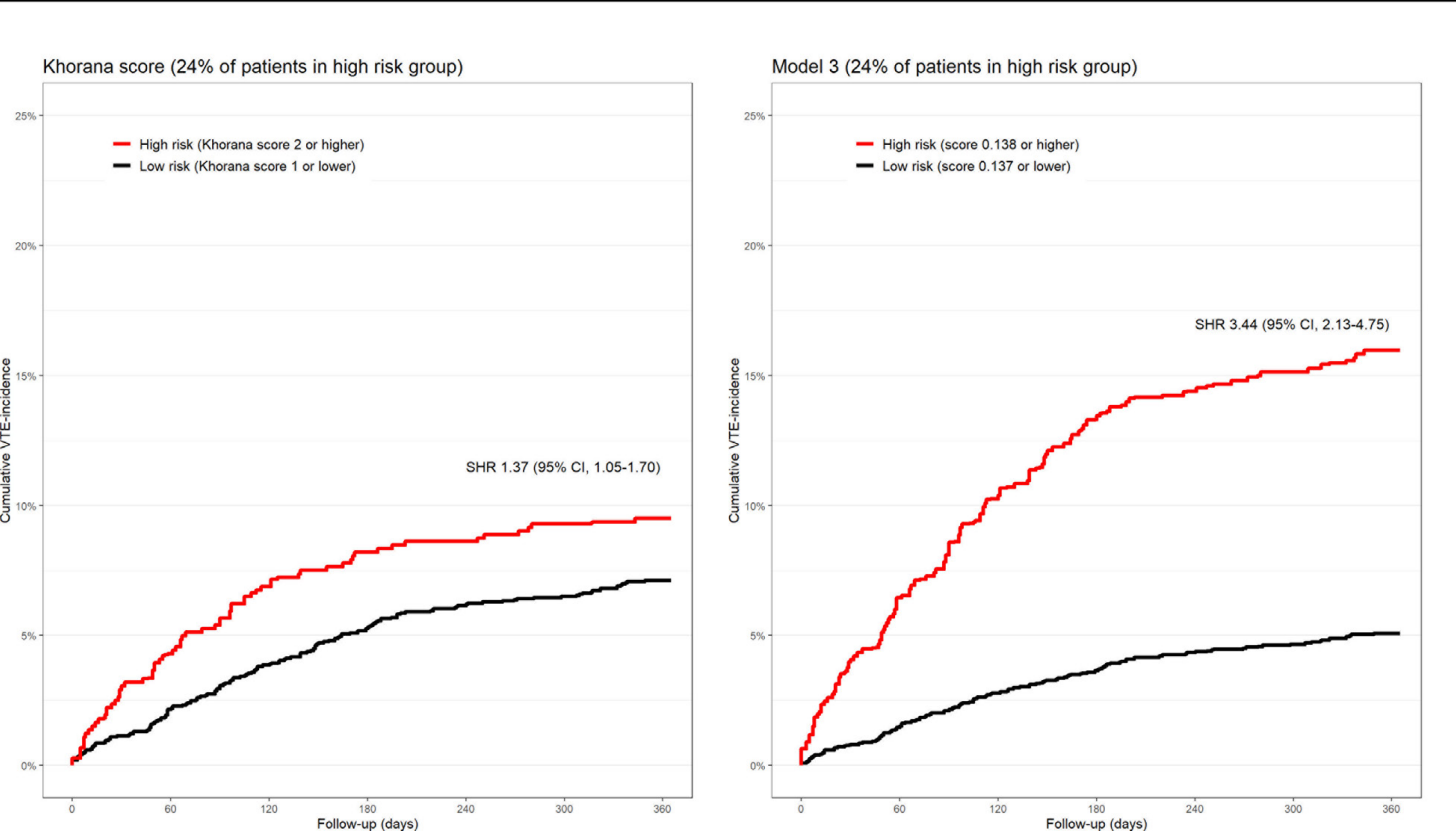
**Cumulative incidence of venous thromboembolism (VTE) in the Khorana risk score and the exploratory combined prediction model 3.** The cumulative VTE incidence for the Khorana score is shown on the **left** and for the combined prediction model 3 on the **right**. SHR indicates subdistribution hazard ratio.

To compare the performance of model 3 with the Khorana score dichotomously, 24% of patients were classified as high risk (using a positivity threshold of 0.138 of the continuous predicted score of model 3) and 76% as low risk. The 12-month cumulative incidence was 16.1% (95% CI, 12.1–20.5) in the high-risk group of model 3 and 5.1% (95% CI, 3.9–6.5) in the low-risk group (subdistribution hazard ratio, 3.44 [95% CI, 2.13–4.75]). The cumulative incidence curves for the risk groups of the Khorana score and of model 3 are shown in Figure 3.

### Secondary Outcome

Of the 3087 cancer patients, 184 (5.9%) developed pulmonary embolism or lower-extremity DVT during 12-month follow-up. The associations between clinical, germline, and somatic mutations and this secondary outcome are shown in Tables S5, S6, and S7 and were comparable to the primary outcome. The optimism-corrected C-indices for the Khorana score and models 1, 2, and 3 were 0.56 (95% CI, 0.51–0.60), 0.67 (95% CI, 0.62–0.70), 0.68 (95% CI, 0.62–0.73), and 0.73 (95% CI, 0.68–0.78), respectively (an overview of model construction and their coefficients is given in Table S8).

### Sensitivity Analyses

Performance of the 3 exploratory models was similar in patients with versus those without anticoagulation use at baseline (Table S9). To complement the bootstrap validation, we performed 10-fold cross-validation across all 27 imputed data sets as an alternative approach to internal validation. The resulting C-indices were 0.66 (95% CI, 0.64–0.68) for model 1, 0.68 (95% CI, 0.63–0.72) for model 2, and 0.77 (95% CI, 0.72–0.82) for model 3 (Table S10).

## Discussion

Using whole-genome tumor sequencing data from a large cohort of patients with metastatic solid cancer, we show that germline and somatic mutations have the potential to improve prediction of cancer-associated VTE. The exploratory prediction models show that the predictive performance of these somatic and germline mutations is maintained conditional on well-established clinical predictors (Figure 2). These models had better discrimination than the clinical Khorana risk score, the current benchmark that is recommended by international guidelines for VTE risk assessment in ambulatory cancer patients.^9–11^

Detailed information on the genetic profile of tumor tissue increasingly aids clinicians in selecting the appropriate cancer treatment for patients.^23,41^ Due to decreasing costs of whole-genome sequencing and the discovery of new molecular targets, this approach may soon become standard care for most cancer types. Using unique, strictly controlled whole-genome data from the tumor and germline, the present study shows that these genetic data, obtained for cancer treatment individualization, can also be utilized to substantially improve the identification of patients at high risk for VTE compared with the Khorana score.^42,43^

Several of the somatic mutations identified in the present study have previously been associated with VTE in the literature, although it is currently unclear whether these represent causal relationships. Two previous studies showed that *CDKN2A* mutations are related to VTE in cancer patients, possibly due to a positive effect on the expression of the prothrombotic proteins podoplanin and tissue factor.^18,44^ The *ELANE* gene is known to be associated with VTE via neutrophil extracellular traps, which promote thrombosis by entrapment of erythrocytes and platelets and activate the intrinsic coagulation pathway.^31,32^ *TBX22*^30^ and *PCDH7*^39^ have previously been reported to be associated with VTE, although the potential mechanism is unknown. Mutations in *SLFN14* (Schlafen Family Member 14) are previously reported to be related to platelet formation and function,^33^ and *PGLYRP4* (Peptidoglycan Recognition Protein 4) to inflammation, 2 potential drivers of hypercoagulability in cancer patients.^34,35^ The reason for the association between the total number of structural variants and VTE may be partly explained by underlying defects in DNA repair pathways leading to genomic instability, which are known features driving aggressive tumor growth and progression.^45^ The identified somatic mutations may inform preclinical studies to better understand the pathophysiology of cancer-associated VTE. The advent of liquid biopsies holds potential to further simplify the use of somatic mutations for VTE prediction.^46^ Future studies may evaluate whether tumor gene expression or tumor single-cell RNA sequencing can further improve prediction.

Besides the somatic mutations, the germline extended polygenic 297-SNP score was also an independent predictor for VTE, which corroborates findings from a recent large-scale analysis using data from the UK Biobank cohort study.^24^ The added value of germline variants for the prediction of cancer-associated VTE was also demonstrated by a recently proposed combined clinical-genetic risk score.^47^ It should be noted that adding the extended 297-SNP score to the model with clinical predictors only slightly increased the C-index from 0.66 (95% CI, 0.62–0.69) to 0.67 (95% CI, 0.62–0.72). The procoagulant effect of germline variants may be relatively small in the presence of strong procoagulant tumor features and clinical predictors. However, as the discriminatory performance of germline variants likely remains stable over time, it may still be a useful predictor, in particular for longer follow-up periods.

This study has several limitations that need to be realized. Most importantly, the number of VTE events was modest (n=237), which limits statistical power and increases the risk of overfitting. The models should be interpreted as exploratory, as we selected somatic mutations with a *P*<0.05 as candidate predictors without correction for multiple testing, thereby accepting an increased risk of false-positive findings. To mitigate this risk as much as possible, we applied a stepwise backward selection strategy, restricted somatic candidates to mutations with a prevalence >2.5%, and used a minimum of 10 events per variable. Moreover, several included mutations have previously been associated with VTE, supporting a biologically plausible link.^18,30–35,39,44^ Second, we were unable to externally validate the findings in an independent cohort, as, to the best of our knowledge, no other studies have evaluated the association between whole-genome tumor mutations and VTE. Third, plasma samples were not available, which precluded evaluation of prognostic biomarkers such as D-dimer levels. Fourth, all patients had metastasized or locally advanced solid tumors, and some were treated with systemic cancer treatment before the biopsy. Therefore, it is unclear whether our results can be extrapolated to those with limited disease and those with a new cancer diagnosis. Fifth, we first applied forward selection of candidate predictors followed by backward selection. While this accounts for correlations among selected variables, predictors with conditional effects, nonlinearities, or interactions may have been missed. Penalized regression methods could address these issues but were beyond the scope of this exploratory study. Last, it can be debated whether 10 events per variable for developing a clinical prediction model is sufficient, since the required sample size is context-specific and also depends on other parameters.^48^ However, we aimed to provide an exploratory prediction model that merely shows the potential of genetic data to improve prediction of cancer-associated VTE rather than a model that can directly be applied in clinical practice.

The main strength of this study includes the availability of unique tumor whole-genome sequencing data together with germline genetic data and clinical data. This allowed us to evaluate the value of genetic information on top of clinical predictors. Patients were enrolled in 12 secondary and tertiary Dutch cancer centers which improved sample diversity and generalizability. Performing central adjudication in our study minimized the risk of bias in outcome assessment, enhancing the reliability and validity of the results.

This study shows that whole-genome somatic and germline variants are associated with VTE in cancer patients, and somatic tumor mutations can improve prediction of this burdensome complication. This can potentially enhance the use of pharmacological thromboprophylaxis. In the near future, genetic data acquired at the time of cancer diagnosis may therefore not only guide selection of cancer treatment but also result in an individualized strategy for prevention of VTE in the oncology population. Given the exploratory nature of our analyses and the absence of external validation, further studies are warranted to confirm these findings and assess their clinical utility.

## Article Information

### Acknowledgments

This publication and research are made possible by the data provided by the Hartwig Medical Foundation and Center of Personalized Cancer Treatment (CPCT). The authors acknowledge all CPCT-02 (Center for Personalized Cancer Treatment-02) participants, the individual Dutch cancer centers for their cooperation, and the Hartwig Medical Foundation.

### Disclosures

Dr van Laarhoven reports consulting or advisory roles for Amphera, AstraZeneca, BeiGene, Bristol Myers Squibb (BMS), Daiichi-Sankyo, Dragonfly, Eli Lilly, MSD, and Servier; research funding and medication supply from Bayer, BMS, Celgene, Janssen, Incyte, Eli Lilly, MSD, Nordic Pharma, Philips, Roche, and Servier; speaker roles for Astellas, Benecke, Daiichi-Sankyo, JAAP, Medtalks, Novartis, and Travel Congress Management BV; and travel support from AstraZeneca. Dr de Vos reports research funding and medication supply from BMS, Ipsen, Johnson & Johnson, Novartis, Pfizer, Roche, Servier, and Foundation STOPbraintumors.org. Dr Westgeest reports honoraria from Merck. Dr Beerepoot reports consulting roles for Servier, Ipsen, and Medtalks; and is a board member of cieBOM NVMO. Dr de Vos-Geelen reports consulting roles for Amgen, AstraZeneca, MSD, Pierre Fabre, and Servier; and institutional research funding from Servier. Dr Labots reports consulting/advisory roles for BMS and MSD; and speaker fees for BMS and Janssen (fees paid to institution). Dr Hamberg reports consulting or advisory fees for Astellas, MSD, Pfizer, AstraZeneca, BMS, and Ipsen. Dr Robbrecht reports consulting or speaker fees from MSD, Merck AG, Pfizer, and AstraZeneca. Dr Steeghs reports consulting or advisory roles for Boehringer Ingelheim, Ellipses Pharma, GlaxoSmithKline, Incyte, and Luszana; and institutional research funding from Abbvie, Actuate Therapeutics, Amgen, Array, Ascendis Pharma, AstraZeneca, Bayer, Blueprint Medicines, Boehringer Ingelheim, BridgeBio, Bristol Myers Squibb, Cantargia, CellCentric, Cogent Biosciences, Crescendo Biologics, Cytovation, Deciphera, Dragonfly, Eli Lilly, Exelixis, Genentech, GlaxoSmithKline, IDRx, Immunocore, Incyte, InteRNA, Janssen, Kinnate Biopharma, Kling Biotherapeutics, Lixte, Luszana, Merck, MSD, Merus, Molecular Partners, Navire Pharma, Novartis, Numab Therapeutics, Pfizer, Relay Pharmaceuticals, Revolution Medicines, Roche, Sanofi, Seattle Genetics, and Takeda. All payments were made to the Netherlands Cancer Institute. Dr van Es reports advisory board and speaker fees from BMS, Bayer, and LEO Pharma; all fees were transferred to his institute. The other authors report no conflicts.

### Supplemental Material

Supplemental Methods

Tables S1–S10

Figure S1

References 49–55

## Supplementary Material
