# Supplementary material for: Tumor Whole-Genome Sequencing for Prediction of Venous Thromboembolism in Patients With Metastasized Solid Cancer
Source: Circ Genom Precis Med. 2026 Apr 10;19(3):e005182. doi: 10.1161/CIRCGEN.124.005182 (PMC13263043; doi:10.1161/CIRCGEN.124.005182)
Supplement: Supplementary file 1 [file hcg-19-e005182-s001.pdf]

SUPPLEMENTAL MATERIAL

SUPPLEMENTAL METHODS

The CPCT-02 cohort study

The CPCT-02 study is a prospective multicenter cohort study in which patients with any type of metastatic solid cancer were enrolled in case of an intended new course of systemic cancer therapy. The goal of CPCT-02 was cancer treatment individualization based on genomic tumor profiling and its design and rationale were discussed in full detail previously.[23] A core needle biopsy was collected from a metastatic tumor lesion (at least 30% histological tumor cellularity required) for whole-genome sequencing, concurrently with a tube of whole blood for whole-genome sequencing of germline DNA. Tumor tissue and whole blood were sequenced at a median depth of 106× and 38×, respectively, using next generation sequencing with the Illumina platform (Illumina, v.2.17 to v.2.20). Samples were aligned against the GRCh37 reference genome and customized software was developed and employed to inventory germline and somatic mutations, microsatellite instability status (MSI) and homologous repair deficiency (HRD) status, as previously detailed by Priestley *et al.*[23] Samples with an absence of somatic variants, tumor purity <20% (PURity & PLoidy Estimator; PURPLE), and high GC-bias were excluded, resulting in a cohort of high-quality samples. The study protocol was approved by the medical ethical committee of the University of Utrecht, and all patients provided written informed consent for whole-genome sequencing and sharing of data for research purposes.

For the present analysis, additional data on patient characteristics and thrombotic outcomes were retrospectively collected for CPCT-02 study participants between January 2019 and February 2021 from their electronic patient records in twelve academic and non-academic Dutch cancer centers using an electronic standardized case report forms. This study was approved by the institutional review boards of participating centers and the medical ethical committee of the University of Rotterdam (MEC2018-1468). Individuals with ≤1 day follow-up, samples with a sex discrepancy, and samples with a heterozygosity rate more than three standard deviations from the mean were excluded using PLINK software. For related samples with a kinship value >0.354 (calculated with KING v.2.0 software), one of the samples was removed, preferably maintaining the case with an outcome event. All ethnicities were retained in the cohort as we aimed to evaluate clinical and genetic predictors in a cohort representative for the full Dutch population.

Outcomes

The primary outcome was VTE occurring from study enrollment up until 12 months of follow-up. VTE was defined as the composite of pulmonary embolism, proximal or distal deep-vein thrombosis of the lower or upper extremities, splanchnic vein thrombosis, and cerebral vein thrombosis. The secondary outcome was a stricter definition of VTE, defined as pulmonary embolism or lower-

extremity DVT. Both symptomatic as incidentally detected events were included. All possible VTE events were verified by two clinicians (N.A.M. and F.I.M.) by carefully assessing the corresponding radiologic imaging reports. Disagreements were resolved by consultation of a third clinician (N.v.E).

Clinical predictors

Candidate clinical predictors were selected based on available literature and included age (continuous),[2] sex,[2] cancer type, hemoglobin level (continuous),[28] leukocyte count (continuous),[28] platelet count (continuous),[28] body mass index (BMI) (continuous),[28] Eastern Cooperative Oncology Group (ECOG) performance score (≥2 vs lower),[49] use of platinum-based chemotherapy,[50] use of gemcitabine,[50] and previous VTE.[2] Cancer types were categorized using the Khorana score classification, which was modified based on VTE incidences observed in a recent large population-based cohort study as follows:[2,28] very high risk for VTE (2 points; gastric cancer, pancreatic cancer, liver cancer, of cholangiocarcinoma), high risk for VTE (1 point; lung cancer, lymphoma, gynecological, testicular cancer, renal cancer, bladder cancer, gastro-esophageal or esophageal cancer, colorectal cancer, multiple myeloma, and brain cancers other than glioblastoma), and low risk for VTE (0 points; other cancer types) (Supplemental Table 2 for classification). Continuous values were transformed, where appropriate, to approach normal distributions. The crude association between candidate clinical predictors and VTE was assessed by calculating univariable hazard ratios (HR) with a Cox proportional regression model in the complete case dataset. The proportional hazards assumption was assessed using Schoenfeld residual-based tests (cox.zph function, survival package in R).

Germline variants and VTE risk

Germline variants were evaluated in a complete case analysis in patients with available genetic data who passed the genetic quality checks (Figure 1). Based on a recent comparison of polygenic risk scores in 36,150 cancer patients of the UK biobank cohort,[24] we chose to evaluate the composite of three single nucleotide polymorphisms (SNP) explaining blood group type O (rsrs8176719, rs505922, and rs8176746) as described earlier[51] and the extended 297-SNP polygenic score as candidate predictors.[17] The latter combines a continuous polygenic risk score of 297 common variants with factor V Leiden and prothrombin gene mutations. If needed, effect alleles for non-ambiguous SNPs of the polygenic risk score were aligned by strand flipping, and for ambiguous SNPs by frequency analysis. Unknown genotype calls were set to the reference allele. Discrimination of the extended 297-SNP score was evaluated by calculating the HR and the C-index with 95% CI by bootstrapping with 250 samples. A Cox regression model was used to evaluate the predictive performance of the germline predictors.

Somatic mutations in tumor tissue and VTE risk

The association between somatic mutations and VTE was evaluated in Cox regression models using a complete case analysis. Per sample, we considered genes to be somatically mutated when they harbored one or multiple protein-coding mutations, disruptive structural variants, or when genes were present within recurrent amplification and/or deletion peaks as detected by GISTIC2.[52] For the univariable analysis, only genes with a mutational prevalence of >1.5% (n>34) were explored. For incorporation in the explorative prediction models only those with a prevalence of >2.5% (n>56).

Additionally, the associations between several tumor characteristics and VTE were evaluated including the presence of microsatellite instability, homologous recombination deficiency as predicted by CHORD[40], total number of structural variants (log continuous), whole genome duplication, genome ploidy (continuous), and tumor mutational burden (log continuous; number of Single Nucleotide Variants, Insertion/Deletions and Multi-nucleotide variants per 1Mbp). All analyses on tumor somatic mutations were adjusted for cancer type using the VTE risk groups specified in Supplemental Table 2.

Imputation and multivariable prediction models

The initial dataset included a subset of patients with clinical data but missing genetic data. Reasons for missing tumor or germline genetic data included the inability to obtain a biopsy or insufficient tumor cellularity. For the construction of multivariable prediction models, missing data for the significant mutations, as identified in the previous complete-case analyses, were imputed by multiple imputation by chained equations (MICE), assuming that data were missing at random, by using 10 iterations and 27 datasets.[53,54]Given the 237 VTE events that occurred during follow-up, a maximum of 24 candidate predictors were pragmatically selected for prediction model construction (~10 events per variable). The 7 strongest significant clinical risk factors (8 degrees of freedom), together with the germline polygenic risk score (1 degree of freedom), an a total of 15 mutational tumor characteristics or somatic mutations with a prevalence of ≥2.5% (23 degrees of freedom) were selected as candidate predictors, based on the lowest P-value in the univariable complete case analyses (total 24 degrees of freedom).

We built three full multivariable Cox regression models consecutively. In the first model, only the 7 clinical predictors were included as candidate predictors. In the second model, the same 7 clinical candidate predictors were evaluated together with the germline extended 297-SNP score. The third model combined the 7 clinical candidate predictors, the germline extended 297-SNP score, and the selected 15 tumor somatic mutations or tumor characteristics. For all three models, a stepwise backward selection approach was used to arrive at the final model, in which variables with a P-value <0.157 (Akaike criterion) were retained. During backward selection, models were compared between

the steps using the likelihood ratio test. Models and coefficients were estimated in each imputed dataset and pooled by the median P rule method.[55]

Internal validation and model evaluation

The C-index was calculated for all constructed models with 95% confidence intervals based on DeLong's method. Outcomes were adjusted for optimism by multiplying the model coefficients and C-indices with the median slopes that were obtained by validating the models with all their candidate predictors in the 27 imputed datasets, each with 250 bootstrap samples using the *rms* package. In addition to internal validation by bootstrapping, we also internally validated all prediction models using 10-fold cross-validation within each of the 27 imputed datasets. The C-indices and corresponding standard errors were computed using the yardstick package in R and were pooled using Rubin's rules.

Calibration of the third model was made clear by plotting the observed cumulative VTE incidence versus the estimated incidence in deciles.

The adjusted third prediction model was compared to that of the Khorana score, the current benchmark prediction score.[28] our dataset, 24% of patients had a Khorana score 2 or higher (considered high risk). To compare discrimination with the dichotomous score, a positivity threshold was selected for the third model by which also 24% of patients were classified as high risk. The Fine and Gray method, which accounts for death as competing risk for VTE, was used to construct time-to-event curves and to calculate the cumulative 12-month VTE incidence in both risk groups, after pooling results in the imputed datasets using Rubin's rule.[25]

All analyses were performed in R software (R Foundation for Statistical Computing, Vienna, Austria; https://www.R-project.org/), in particular using packages *psfmi*, *mice*, and *rms*.

SUPPLEMENTAL TABLES

Supplemental Table 1. Tripod checklist

Section/Topic	n	Checklist Item	Page
**Title and abstract**			
Title	1	Identify the study as developing and/or validating a multivariable prediction model, the target population, and the outcome to be predicted.	√
Abstract	2	Provide a summary of objectives, study design, setting, participants, sample size, predictors, outcome, statistical analysis, results, and conclusions.	√
**Introduction**			
Background and objectives	3a	Explain the medical context (including whether diagnostic or prognostic) and rationale for developing or validating the multivariable prediction model, including references to existing models.	√
	3b	Specify the objectives, including whether the study describes the development or validation of the model or both.	√
**Methods**			
Source of data	4a	Describe the study design or source of data (e.g., randomized trial, cohort, or registry data), separately for the development and validation data sets, if applicable.	√
	4b	Specify the key study dates, including start of accrual; end of accrual; and, if applicable, end of follow-up.	√
Participants	5a	Specify key elements of the study setting (e.g., primary care, secondary care, general population) including number and location of centres.	√
	5b	Describe eligibility criteria for participants.	√
	5c	Give details of treatments received, if relevant.	-
Outcome	6a	Clearly define the outcome that is predicted by the prediction model, including how and when assessed.	√
	6b	Report any actions to blind assessment of the outcome to be predicted.	-
Predictors	7a	Clearly define all predictors used in developing or validating the multivariable prediction model, including how and when they were measured.	√

	7b	Report any actions to blind assessment of predictors for the outcome and other predictors.	-
Sample size	8	Explain how the study size was arrived at.	√
Missing data	9	Describe how missing data were handled (e.g., complete-case analysis, single imputation, multiple imputation) with details of any imputation method.	√
Statistical analysis methods	10a	Describe how predictors were handled in the analyses.	√
	10b	Specify type of model, all model-building procedures (including any predictor selection), and method for internal validation.	√
	10d	Specify all measures used to assess model performance and, if relevant, to compare multiple models.	√
Risk groups	11	Provide details on how risk groups were created, if done.	√
**Results**			
Participants	13a	Describe the flow of participants through the study, including the number of participants with and without the outcome and, if applicable, a summary of the follow-up time. A diagram may be helpful.	√
	13b	Describe the characteristics of the participants (basic demographics, clinical features, available predictors), including the number of participants with missing data for predictors and outcome.	√
Model development	14a	Specify the number of participants and outcome events in each analysis.	√
	14b	If done, report the unadjusted association between each candidate predictor and outcome.	√
Model specification	15a	Present the full prediction model to allow predictions for individuals (i.e., all regression coefficients, and model intercept or baseline survival at a given time point).	√
	15b	Explain how to the use the prediction model.	√
Model performance	16	Report performance measures (with CIs) for the prediction model.	√
**Discussion**			
Limitations	18	Discuss any limitations of the study (such as nonrepresentative sample, few events per predictor, missing data).	√

Interpretation	19b	Give an overall interpretation of the results, considering objectives, limitations, and results from similar studies, and other relevant evidence.	√
Implications	20	Discuss the potential clinical use of the model and implications for future research.	√
**Other information**			
Supplemental information	21	Provide information about the availability of Supplemental resources, such as study protocol, Web calculator, and data sets.	√
Funding	22	Give the source of funding and the role of the funders for the present study.	√

Supplemental Table 2. Classification of cancer types according to risk of venous thromboembolism.*

**Points**	**Risk group**	**Cancer type**
2	Very high risk	Gastric cancer<br>Pancreatic cancer<br>Glioblastoma<br>Cholangiocarcinoma<br>Liver cancer
1	High risk	Lung cancer<br>Lymphoma<br>Gynecological<br>Testicular cancer<br>Bladder cancer<br>Gastro-esophageal cancer<br>Esophageal cancer<br>Renal cancer<br>Colorectal cancer (including appendix)<br>Brain cancer other than glioblastoma

*Classification based on the Khorana score with addition of several cancer types, based on the incidence rate of venous thromboembolism per 1,000 person years during 6 months follow-up in a population-based study of Danish Cancer patients.[2] Glioblastoma, cholangiocarcinoma and liver cancer added to the very high risk group, and gastro-esophageal cancer, esophageal cancer, colorectal cancer, renal cancer, brain cancer other than glioblastoma, and multiple myeloma added to high risk group.

Supplemental Table 3. Whole-genome somatic mutations in tumor tissue associated with venous thromboembolism during 12 month-follow-up.

**Gene**	**Hazard ratio (95% confidence interval)**	**P-value**	**Mutation prevalence (%)**
POLR2E	3.425 (1.684-6.967)	0.00068	1.61
PALM	3.732 (1.743-7.987)	0.0007	1.57
TBX22	2.47 (1.402-4.353)	0.00176	3.14
ELANE	3.221 (1.511-6.867)	0.00246	1.52
SLFN14	2.833 (1.392-5.767)	0.00409	1.88
POTEF	2.655 (1.356-5.199)	0.00439	2.06
PGLYRP4	3.288 (1.448-7.468)	0.00446	1.48
CDKN2A	1.569 (1.147-2.144)	0.00479	28.41
OR4L1	2.72 (1.337-5.536)	0.00577	1.84
PCDH7	2.466 (1.299-4.681)	0.00579	2.64
FCRL4	2.56 (1.307-5.013)	0.00612	2.42
KCNK10	2.65 (1.303-5.389)	0.00713	1.84
COL22A1	0.359 (0.168-0.765)	0.00799	9.54
ADAMTS20	1.936 (1.188-3.154)	0.00804	6
ZC3H12C	2.588 (1.273-5.264)	0.00865	2.15
CSMD3	0.512 (0.31-0.846)	0.00896	17.65
OR4K1	2.442 (1.248-4.78)	0.00914	2.42
CYP3A43	2.72 (1.276-5.799)	0.00958	1.7
RNF123	2.849 (1.259-6.444)	0.01196	1.61
PLEC	0.352 (0.156-0.795)	0.01200	8.33
IARS2	2.635 (1.236-5.616)	0.01208	1.61

WDR78	2.342 (1.197-4.585)	0.01299	2.11
FRMPD4	0.172 (0.043-0.692)	0.01327	5.42
WDR64	2.235 (1.18-4.234)	0.01359	2.82
SRGAP2	2.441 (1.2-4.962)	0.01372	2.11
BRINP2	2.216 (1.169-4.202)	0.01478	3.05
NUP133	2.559 (1.201-5.453)	0.01489	1.57
ANKMY1	2.399 (1.18-4.879)	0.01570	2.11
ANKK1	2.395 (1.176-4.876)	0.01604	2.37
DCST1	2.657 (1.176-6.004)	0.01877	1.7
PCDHGA4	2.145 (1.132-4.064)	0.01930	2.87
XKR3	2.224 (1.134-4.359)	0.01996	2.51
ESRRG	2.451 (1.15-5.225)	0.02021	1.93
BNIPL	2.447 (1.149-5.215)	0.02041	1.93
HCN2	2.841 (1.163-6.941)	0.02198	1.52
TNPO2	2.406 (1.128-5.13)	0.02305	1.75
TMEM259	2.394 (1.124-5.102)	0.02371	1.84
ASMT	0.2 (0.05-0.807)	0.02376	4.61
MIER2	2.393 (1.122-5.102)	0.02390	2.02
KDM6A	0.319 (0.118-0.86)	0.02402	6.5
LRRC4B	2.39 (1.119-5.104)	0.02436	1.88
LRP1B	0.603 (0.388-0.938)	0.02469	19.8
CBARP	2.37 (1.112-5.05)	0.02537	1.93
ASH1L	1.952 (1.085-3.51)	0.02556	4.03
DNHD1	0.107 (0.015-0.762)	0.02567	4.44
LRRK2	1.867 (1.079-3.231)	0.02573	4.88

ADAMTS12	1.772 (1.071-2.932)	0.02597	5.91
CEP135	2.234 (1.098-4.546)	0.02651	2.2
OR4K5	2.356 (1.105-5.023)	0.02655	1.97
OR4K13	2.228 (1.096-4.532)	0.02697	1.97
ZNF804A	0.277 (0.088-0.868)	0.02762	5.65
CLK2	2.498 (1.106-5.644)	0.02765	1.57
OR4K2	2.128 (1.086-4.167)	0.02768	2.33
MISP	2.497 (1.103-5.652)	0.02808	1.84
FREM2	0.329 (0.122-0.888)	0.02813	6.59
PLXNC1	2.337 (1.095-4.987)	0.02823	1.97
FHIT	1.681 (1.055-2.679)	0.02895	7.03
LAMB2	2.314 (1.086-4.931)	0.02976	2.2
DNAH6	1.677 (1.05-2.678)	0.03030	7.89
OR4K15	2.302 (1.08-4.909)	0.03089	1.75
SLCO4A1	2.188 (1.074-4.459)	0.03113	2.15
IRF2BP2	2.442 (1.081-5.514)	0.03173	1.57
CDH9	0.337 (0.125-0.91)	0.03180	5.96
SLC25A6	0.116 (0.016-0.831)	0.03201	3.99
AXDND1	2.171 (1.067-4.419)	0.03252	2.64
FRG2C	2.427 (1.074-5.484)	0.03296	1.75
MYO15A	0.289 (0.092-0.907)	0.03332	5.91
ANO1	1.733 (1.044-2.876)	0.03348	7.21
BHLHE41	2.435 (1.072-5.533)	0.03352	1.7
TNRC18	0.29 (0.093-0.908)	0.03361	5.29
MYRIP	2.418 (1.07-5.464)	0.03382	1.79

CHST9	2.269 (1.064-4.841)	0.03397	1.88
GPLD1	2.412 (1.068-5.447)	0.03414	1.52
SBF2	2.152 (1.058-4.377)	0.03428	2.06
SYT16	2.264 (1.062-4.826)	0.03434	1.75
DCAF8L2	0.222 (0.055-0.895)	0.03439	4.17
RORB	2.268 (1.06-4.849)	0.03475	2.02
SHOX	0.223 (0.055-0.899)	0.03492	4.21
MBNL3	2.39 (1.057-5.405)	0.03644	1.52
P2RY8	0.123 (0.017-0.876)	0.03647	3.94
DHRSX	0.123 (0.017-0.88)	0.03687	3.72
ZBTB7B	2.242 (1.05-4.788)	0.03708	1.93
ADAM7	0.448 (0.21-0.955)	0.03753	8.51
PRKX	0.124 (0.017-0.887)	0.03761	3.67
IL3RA	0.229 (0.057-0.924)	0.03841	4.17
MTOR	0.229 (0.057-0.925)	0.03853	4.48
ARSF	0.23 (0.057-0.927)	0.03875	4.35
SPTBN4	0.301 (0.096-0.942)	0.0391	5.24
ADGRF5	2.03 (1.036-3.979)	0.03923	3.09
NUP214	2.106 (1.036-4.283)	0.03968	2.15
PDE6C	2.342 (1.037-5.29)	0.04067	1.61
LRP2	0.477 (0.234-0.969)	0.04080	8.65
GRM2	2.199 (1.031-4.69)	0.04136	1.79
PSD3	0.428 (0.189-0.967)	0.04141	7.35
ZNF530	2.529 (1.037-6.171)	0.04146	1.52
CECR2	1.948 (1.026-3.7)	0.04156	3.23

IL6R	2.332 (1.032-5.268)	0.04169	1.48
FOCAD	0.397 (0.163-0.966)	0.04173	6.81
KIAA1109	0.306 (0.098-0.959)	0.04215	5.33
CHD6	0.13 (0.018-0.93)	0.04215	4.08
ERICH1	0.429 (0.19-0.971)	0.04219	7.21
LRRC4C	0.131 (0.018-0.934)	0.04250	3.99
ADGRB3	1.686 (1.018-2.793)	0.04255	6.23
IGSF9	1.933 (1.02-3.664)	0.04322	3
CDH1	0.308 (0.098-0.965)	0.04327	5.78
TBK1	2.501 (1.026-6.093)	0.04370	1.48
PTK2	0.239 (0.059-0.963)	0.04408	4.66
PRMT8	2.309 (1.022-5.215)	0.04409	1.75
MID1	0.134 (0.019-0.955)	0.04488	3.45
EGF	2.174 (1.018-4.642)	0.04491	2.06
ACHE	2.165 (1.016-4.615)	0.04542	1.84
SYT14	2.298 (1.017-5.191)	0.04544	1.52
FGA	2.066 (1.014-4.209)	0.04559	2.73
KMT2B	0.364 (0.135-0.981)	0.04570	5.69
ARSL	0.135 (0.019-0.965)	0.04598	3.54
SCFD1	2.295 (1.014-5.194)	0.04620	1.75
SLC45A4	0.135 (0.019-0.967)	0.04621	3.67
INTS6L	2.058 (1.012-4.184)	0.04633	2.24
AKAP17A	0.314 (0.1-0.983)	0.04672	4.39
CACNA1F	0.314 (0.1-0.985)	0.04701	4.88
CRNN	2.284 (1.009-5.168)	0.04742	1.93

DDX54	2.281 (1.009-5.157)	0.04746	1.57
OR2B11	2.461 (1.009-6.003)	0.04780	1.61
XKR6	0.439 (0.194-0.993)	0.04809	7.21
R3HDM4	2.272 (1.006-5.13)	0.04827	1.52
BCOR	0.368 (0.136-0.993)	0.04846	5.24
MED16	2.14 (1.004-4.56)	0.04875	2.11
TLR7	0.139 (0.019-0.993)	0.04923	3.72
GRAMD1B	2.035 (1.001-4.138)	0.04983	2.24

Supplemental Table 4. Variables of the three explorative prediction models

	**Model 1**	**Model 2**	**Model 3**
Evaluated clinical variables	Cancer type reference group<br>Cancer type high VTE risk group<br>Cancer type very high VTE risk group<br>Leukocyte count (per log increase)<br>Platinum based chemotherapy (yes vs no)<br>ECOG (≥2 vs lower)<br>Prior VTE<br>Log2 platelet count (per log2 increase)<br>Hemoglobin count^2 (per count^2 increase)	Cancer type reference group<br>Cancer type high VTE risk group<br>Cancer type very high VTE risk group<br>Leukocyte count (per log increase)<br>Platinum based chemotherapy (yes vs no)<br>ECOG (≥2 vs lower)<br>Prior VTE<br>Log2 platelet count (per log2 increase)<br>Hemoglobin count^2 (per count^2 increase)	Cancer type reference group<br>Cancer type high VTE risk group<br>Cancer type very high VTE risk group<br>Leukocyte count (per log increase)<br>Platinum based chemotherapy (yes vs no)<br>ECOG (≥2 vs lower)<br>Prior VTE<br>Log2 platelet count (per log2 increase)<br>Hemoglobin count^2 (per count^2 increase)
Evaluated germline variables	-	297-extended SNP score (per point increase)	297-extended SNP score (per point increase)
Evaluated whole-genome tumor mutation variables	-	-	Total number of structural variants (continuous, per 1000 increase)<br>TBX22<br>CDKN2A<br>PCDH7<br>COL22A1<br>ADAMTS20<br>CSMD3<br>PLEC<br>FRMPD4<br>WDR64<br>BRINP2<br>PCDHGA4<br>XKR3<br>ASMT<br>KDM6A
Degrees of freedom	8	9	24
Variables included in final model (coefficient)	Cancer type high VTE risk group (0.558)<br>Cancer type very high VTE risk group (0.795)<br>Leukocyte count (0.543)<br>ECOG (0.468)<br>Platinum based chemotherapy (0.367)<br>Prior VTE (0.365)	Cancer type high VTE risk group (0.572)<br>Cancer type very high VTE risk group (0.815)<br>Leukocyte count (0.537)<br>ECOG (0.487)<br>Platinum based chemotherapy (0.355)<br>Prior VTE (0.318)	Cancer type high VTE risk group (0.603)<br>Cancer type very high VTE risk group (0.758)<br>Leukocyte count (0.451)<br>ECOG (0.421)<br>Platinum based chemotherapy (0.387)<br>Prior VTE (0.327)

		297-extended SNP score (0.6)	Total number of structural variants (-0.000466)<br>TBX22 (0.735)<br>CDKN2A (0.277)<br>PCDH7 (0.544)<br>COL22A1 (-0.524)<br>ADAMTS20 (0.496)<br>CSMD3 (-0.486)<br>PLEC (-0.553)<br>FRMPD4 (-0.742)<br>WDR64 (0.316)<br>BRINP2 (0.233)<br>PCDHGA4 (0.238)<br>XKR3 (0.206)<br>ASMT (-0.355)<br>KDM6A (-0.423)

* The prognostic index can be calculated by multiplying all variables of the model with its coefficient.

Abbreviations; CI, confidence interval; ECOG, Eastern Cooperative Oncology Group; VTE, venous thromboembolism

Supplemental Table 5. Association between clinical predictors and VTE during 12 months follow-up, univariable cox regression analysis, sensitivity analysis with stricter definition of VTE.

**Clinical variable**	**Hazard ratio (95% CI)**	**p-value**
Cancer type		
Reference group	-	-
High VTE risk (lung, gynecological, lymphoma, bladder, testicular, colorectal, esophagus, renal, brain other than glioblastoma, multiple myeloma)	2.18 (1.58-3.01)	$2.3*10^{-6}$
Very high VTE risk (pancreas, stomach, glioblastoma, hepatobiliary)	2.66 (1.71-4.14)	$1.5*10^{-5}$
Log leukocyte count (per log increase)	2.46 (1.73-3.51)	$6,01*10^{-7}$
Use of platinum based chemotherapy (yes vs no)	2.16 (1.59-2.92)	$6,43*10^{-7}$
ECOG performance status ≥2 vs lower	2.27 (1.41-3.66)	$7.7*10^{-4}$
Log2 platelet count	1.3 (0.98-1.72)	0.065
Age^2 in years	1 (0.9998-1)	0.19
Prior venous thromboembolism	1.33 (0.86-2.06)	0.20
Hemoglobin $count^2$	0.995 (0.99-1.004)	0.28
Log2 body-mass index	1.17 (0.65-2.09)	0.61
Use of gemcitabine chemotherapy (yes vs no)	0.977 (0.50-1.91)	0.95
Sex (male vs female)	0.997 (0.75-1.3)	0.98

Abbreviations; CI, confidence interval; ECOG, Eastern Cooperative Oncology Group; VTE, venous thromboembolism

^{2} squared

Supplemental Table 6. Association between tumor genetics and venous thromboembolism during 12 months follow-up, cox regression analysis, sensitivity analysis with stricter definition of VTE.

	**Hazard ratio (95% CI)**	**p-value**	**Prevalence of mutation (%)**
**Top 10 whole genome tumor mutations associated with VTE**			
POLR2E	3.93 (1.83-8.41)	0.00043	1.61
OR4K1	3.24 (1.65-6.39)	0.00066	2.42
ZC3H12C	3.43 (1.67-7.00)	0.00074	2.15
PALM	4.06 (1.78-9.26)	0.00085	1.57
BRINP2	2.92 (1.53-5.58)	0.00113	3.05
LRRK2	2.48 (1.42-4.32)	0.00138	4.88
ELANE	3.56 (1.57-8.07)	0.00242	1.52
DCST1	3.53 (1.55-8.00)	0.00259	1.7
OR4K2	2.82 (1.43-5.55)	0.00272	2.33
OR4K13	2.96 (1.45-6.06)	0.00292	1.97
**Tumor mutational characteristics**			
Total number of structural variants (continuous, per 1000 increase)*	1.22 (1.07-1.38)	0.002	-
Whole genome duplication (binary)	1.11 (1.02-1.21)	0.02	-
Total tumor mutational burden (continuous, per log increase)	1.04 (0.99-1.07)	0.054	-
Homologous recombination deficiency (CHORD[40])	0. 87 (0.73-1.05)	0.14	-
Genome ploidy (continuous)	1.02 (0.97-1.07)	0.43	-
Microsatellite instability	1.07 (0.78-1.48)	0.68	-

* Natural algorithm

Abbreviations: CI, confidence interval; VTE, venous thromboembolism

Supplemental Table 7. Whole-genome somatic mutations in tumor tissue associated with VTE during 12 month-follow-up, sensitivity analysis using a stricter definition for VTE.

**Gene**	**Hazard ratio (95% confidence interval)**	**p-value**	**Mutation prevalence (%)**
POLR2E	3.927 (1.834-8.411)	0.00043	1.61
OR4K1	3.244 (1.649-6.385)	0.00066	2.42
ZC3H12C	3.426 (1.676-7.004)	0.00074	2.15
PALM	4.065 (1.784-9.264)	0.00085	1.57
BRINP2	2.924 (1.533-5.579)	0.00113	3.05
LRRK2	2.479 (1.421-4.323)	0.00138	4.88
ELANE	3.556 (1.567-8.07)	0.00242	1.52
DCST1	3.526 (1.553-8.003)	0.00259	1.7
OR4K2	2.82 (1.432-5.554)	0.00272	2.33
OR4K13	2.962 (1.449-6.055)	0.00292	1.97
OR4K5	3.138 (1.464-6.725)	0.00328	1.97
OR4L1	3.083 (1.439-6.605)	0.00378	1.84
CLK2	3.324 (1.465-7.542)	0.00406	1.57
CHST9	3.024 (1.41-6.483)	0.00446	1.88
KCNK10	2.947 (1.376-6.308)	0.0054	1.84
RORB	2.929 (1.362-6.296)	0.00592	2.02
ASH1L	2.374 (1.28-4.402)	0.00606	4.03
INTS6L	2.705 (1.324-5.53)	0.00636	2.24
DNAH6	1.991 (1.209-3.278)	0.00682	7.89
ADAMTS20	2.092 (1.222-3.58)	0.00711	6
PTPRN	2.666 (1.304-5.45)	0.0072	2.55

PDE6C	3.043 (1.341-6.903)	0.00776	1.61
PGLYRP4	3.381 (1.375-8.31)	0.00794	1.48
ZEB2	2.361 (1.237-4.508)	0.00923	3.14
C6ORF118	2.45 (1.242-4.831)	0.00972	2.69
ANKK1	2.726 (1.272-5.845)	0.00996	2.37
CRNN	2.939 (1.293-6.68)	0.01008	1.93
IARS2	2.918 (1.287-6.62)	0.01038	1.61
PPFIA4	2.543 (1.244-5.197)	0.01049	2.55
ANKMY1	2.686 (1.255-5.752)	0.01097	2.11
SRGAP2	2.676 (1.25-5.728)	0.01124	2.11
PGLYRP3	3.194 (1.301-7.841)	0.01129	1.61
NUP133	2.88 (1.27-6.53)	0.01135	1.57
PM20D1	2.879 (1.268-6.534)	0.01148	1.75
RMDN1	2.691 (1.249-5.801)	0.0115	2.42
F9	2.511 (1.224-5.149)	0.01199	2.42
TMEM121B	2.83 (1.244-6.441)	0.01315	1.79
CHRNB2	2.775 (1.223-6.294)	0.01462	2.02
DMBT1	2.169 (1.164-4.041)	0.01473	4.66
FLAD1	3.015 (1.233-7.372)	0.01556	1.48
IGSF9	2.299 (1.168-4.524)	0.01599	3
SLC4A1	2.396 (1.171-4.9)	0.01673	2.37
ESRRG	2.71 (1.194-6.149)	0.01708	1.93
EBF3	2.709 (1.194-6.145)	0.01711	1.7
FCRL1	2.717 (1.194-6.187)	0.01725	1.93
ADGRB3	1.925 (1.12-3.308)	0.0178	6.23

CLTCL1	2.498 (1.167-5.346)	0.01841	2.11
TNPO2	2.679 (1.181-6.082)	0.01843	1.75
TMEM259	2.662 (1.174-6.039)	0.01911	1.84
FCRL4	2.484 (1.159-5.323)	0.01926	2.42
CBARP	2.627 (1.158-5.959)	0.02079	1.93
CECR2	2.23 (1.13-4.401)	0.02082	3.23
PRMT9	2.865 (1.171-7.011)	0.02118	1.48
WDR64	2.307 (1.129-4.716)	0.02189	2.82
ZBTB7B	2.611 (1.148-5.939)	0.02204	1.93
ARHGEF2	2.599 (1.146-5.893)	0.02226	1.97
COL21A1	2.126 (1.111-4.066)	0.02266	4.35
CCDC178	2.194 (1.115-4.317)	0.02282	3.32
ADGRF5	2.287 (1.117-4.683)	0.02371	3.09
AVPR1B	2.799 (1.143-6.854)	0.02426	1.52
SLC26A9	2.274 (1.11-4.657)	0.02468	2.78
SOX9	2.272 (1.11-4.647)	0.02468	2.6
PRKAA2	2.798 (1.139-6.871)	0.02478	1.84
EPHA3	2.091 (1.097-3.984)	0.02496	3.94
KDM6A	0.202 (0.05-0.819)	0.02504	6.5
FOCAD	0.205 (0.051-0.831)	0.0264	6.81
SBF2	2.368 (1.106-5.07)	0.02645	2.06
GRAMD1B	2.367 (1.105-5.067)	0.02656	2.24
COL22A1	0.396 (0.174-0.898)	0.02656	9.54
OR2T6	2.759 (1.125-6.767)	0.02665	1.57
TGFBR2	2.256 (1.097-4.639)	0.02695	2.6

OR4K15	2.518 (1.109-5.715)	0.02729	1.75
FRMPD4	0.109 (0.015-0.783)	0.02754	5.42
SYT16	2.509 (1.106-5.693)	0.02774	1.75
OR11L1	2.504 (1.103-5.688)	0.0283	1.75
CSMD3	0.54 (0.31-0.942)	0.02991	17.65
FGA	2.32 (1.081-4.977)	0.0307	2.73
WDR78	2.315 (1.081-4.958)	0.03078	2.11
OR4E2	2.671 (1.093-6.527)	0.03122	1.48
NOTCH2	2.2 (1.074-4.507)	0.03123	3.49
MISP	2.673 (1.091-6.549)	0.03149	1.84
DSG4	2.097 (1.065-4.13)	0.03217	3.72
MYO1A	2.448 (1.079-5.555)	0.03217	2.06
LMX1A	2.444 (1.077-5.548)	0.03258	1.93
LAMB3	2.29 (1.07-4.905)	0.03293	2.46
OR2T4	2.443 (1.074-5.555)	0.03314	1.88
PLEC	0.38 (0.155-0.929)	0.03379	8.33
STRN3	2.428 (1.07-5.513)	0.03392	1.7
ACHE	2.424 (1.068-5.499)	0.03414	1.84
PRDM2	2.278 (1.063-4.878)	0.03417	2.37
TRAF3IP3	2.627 (1.074-6.426)	0.03433	1.57
BCL9L	2.156 (1.055-4.405)	0.03517	2.87
MED16	2.41 (1.062-5.468)	0.03529	2.11
TMTC2	2.407 (1.061-5.458)	0.0355	1.88
CCT8L2	2.16 (1.053-4.433)	0.0357	2.73
HCN2	2.887 (1.063-7.839)	0.03752	1.52

KMT2B	0.231 (0.057-0.936)	0.04003	5.69
CDON	2.548 (1.042-6.232)	0.04038	1.7
TOGARAM1	2.034 (1.032-4.011)	0.04041	3.14
ASMT	0.128 (0.018-0.918)	0.04079	4.61
NXPE4	2.348 (1.035-5.327)	0.04123	2.15
ARFGEF3	2.022 (1.028-3.978)	0.04146	3.58
DCST2	2.534 (1.036-6.201)	0.0417	1.79
XKR3	2.205 (1.028-4.73)	0.04218	2.51
SIK3	2.521 (1.031-6.164)	0.04269	1.57
MBNL3	2.522 (1.03-6.172)	0.04282	1.52
ADORA1	2.519 (1.028-6.17)	0.04333	1.66
PLCB4	2.009 (1.021-3.953)	0.0434	3.67
MYRIP	2.512 (1.027-6.143)	0.04351	1.79
ZNF804A	0.238 (0.059-0.962)	0.04403	5.65
CLCA4	2.316 (1.02-5.255)	0.04464	2.28
PSD3	0.362 (0.134-0.979)	0.04539	7.35
TRIM46	2.491 (1.018-6.093)	0.0455	1.7
FAM135B	0.402 (0.164-0.982)	0.04555	8.38
STK31	2.172 (1.013-4.656)	0.04621	2.91
R3HDM4	2.472 (1.012-6.041)	0.04707	1.52
CYP3A43	2.471 (1.01-6.044)	0.04748	1.7
ERICH1	0.366 (0.135-0.991)	0.04802	7.21
DDX54	2.46 (1.005-6.018)	0.04863	1.57
ADAM7	0.407 (0.167-0.996)	0.04905	8.51
INSC	2.287 (1.002-5.218)	0.04936	1.75

KLHL4	1.912 (1.001-3.653)	0.04976	3.94

Abbreviations; CI, confidence interval; ECOG, Eastern Cooperative Oncology Group; VTE, venous thromboembolism

Supplemental Table 8. Variables of the three explorative prediction models

	**Model 1**	**Model 2**	**Model 3**
Evaluated clinical variables	Cancer type reference group<br>Cancer type high VTE risk group<br>Cancer type very high VTE risk group<br>Leukocyte count (per log increase)<br>Platinum based chemotherapy (yes vs no)<br>ECOG (≥2 vs lower)	Cancer type reference group<br>Cancer type high VTE risk group<br>Cancer type very high VTE risk group<br>Leukocyte count (per log increase)<br>Platinum based chemotherapy (yes vs no)<br>ECOG (≥2 vs lower)	Cancer type reference group<br>Cancer type high VTE risk group<br>Cancer type very high VTE risk group<br>Leukocyte count (per log increase)<br>Platinum based chemotherapy (yes vs no)<br>ECOG (≥2 vs lower)
Evaluated germline variables	-	297-extended SNP score (per point increase)	297-extended SNP score (per point increase)
Evaluated whole-genome tumor mutation variables	-	-	Number of structural variants (per 1000 increase)<br>BRINP2<br>LRRK2<br>ASH1L<br>DNAH6<br>ADAMTS20<br>PTPRN<br>ZEB2<br>C6ORF118<br>PPFIA4<br>IGSF9<br>ADGRB3
Degrees of freedom	5	6	18
Variables included in final model (coefficient)	ECOG (0.641)<br>Leukocyte count (0.665)<br>Platinum chemotherapy (0.476)<br>Cancer type high VTE risk group (0.531)<br>Cancer type very high VTE risk group (0.613)	ECOG (0.636)<br>Leukocyte count (0.656)<br>Platinum chemotherapy (0.457)<br>297-extended SNP score (0.41)<br>Cancer type high VTE risk group (0.532)<br>Cancer type very high VTE risk group (0.619)	ECOG (0.556)<br>Leukocyte count (0.524)<br>Platinum chemotherapy (0.404)<br>297-extended SNP score (0.416)<br>Number of structural variants (-0.000404)<br>BRINP2 (-0.0825)<br>LRRK2 (0.2609)<br>ASH1L (0.279)

			DNAH6 (0.2209)<br>ADAMTS20 (-0.0673)<br>PTPRN (-0.1209)<br>ZEB2 (-0.03)<br>C6ORF118 (0.182)<br>PPFIA4 (0.0721)<br>IGSF9 (0.0635)<br>ADGRB3 (-0.0803)<br>Cancer type high VTE risk group (0.549)<br>Cancer type very high VTE risk group (0.679)

* The prognostic index can be calculated by multiplying all variables of the model with its coefficient.

Abbreviations; CI, confidence interval; ECOG, Eastern Cooperative Oncology Group; VTE, venous thromboembolism

Supplemental Table 9. Performance of exploratory models in the total study population and in the study population without anticoagulants at baseline.

Prediction Model	Total Study population (C-index, 95% CI)	Without Anticoagulants (C-index, 95% CI)
Model 1	0.66 (0.62–0.69)	0.65 (0.62–0.69)
Model 2	0.67 (0.62–0.72)	0.67 (0.62–0.72)
Model 3	0.77 (0.72–0.81)	0.77 (0.72–0.81)

Abbreviations: CI, confidence interval

Supplemental Table 10. Performance the exploratory models when using cross-validation with 10 folds in the 27 imputed datasets

Prediction Model	Total Study Group (C-index, 95% CI)
Model 1	0.66 (0.64–0.68)
Model 2	0.68 (0.63–0.72)
Model 3	0.77 (0.72–0.82)

Abbreviations: CI, confidence interval

SUPPLEMENTAL FIGURES

Supplemental Figure 1. Calibration plot of the third prediction model, observed versus estimated cumulative VTE incidence during 12 months follow-up.

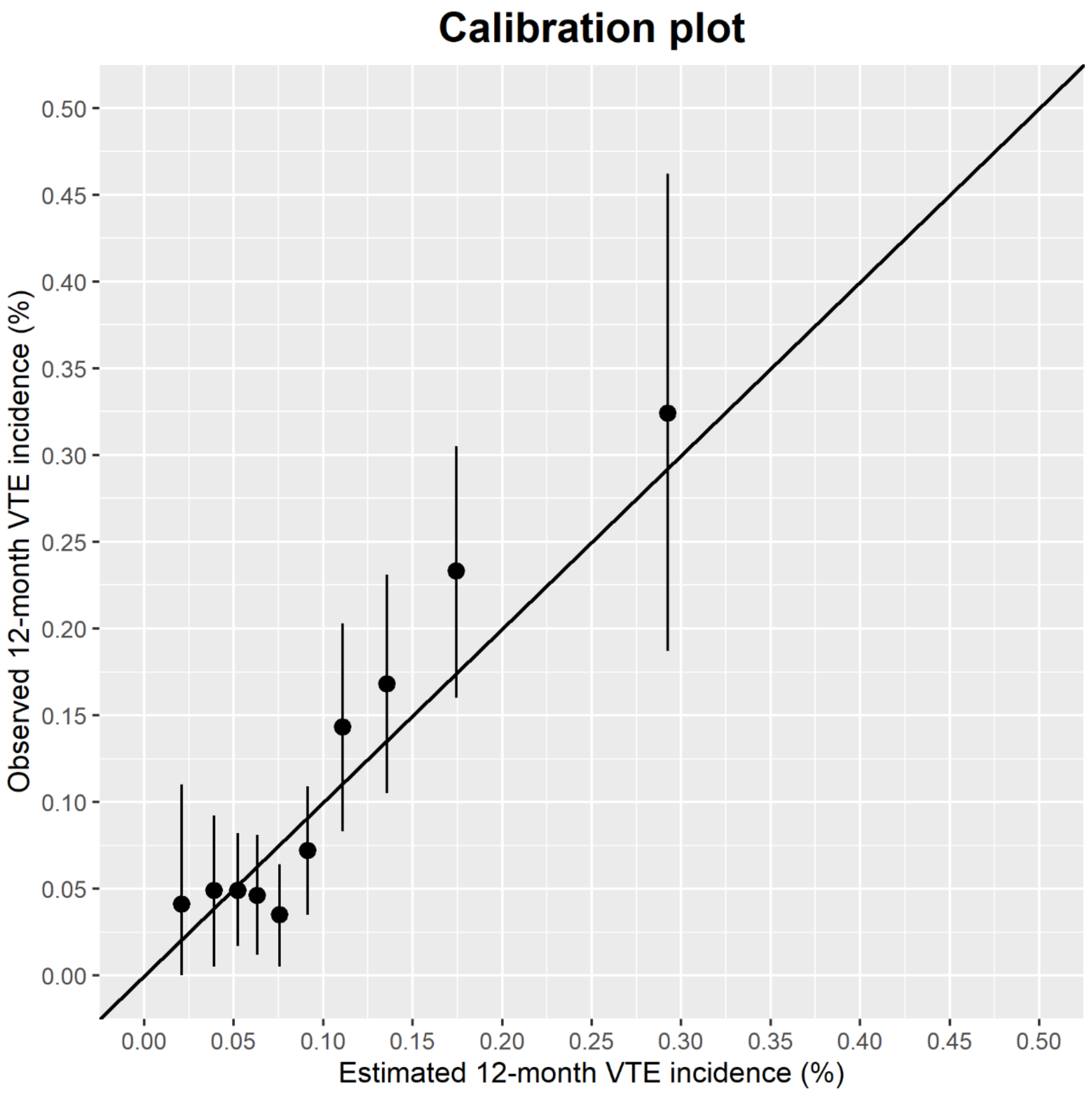

Abbreviations: VTE, venous thromboembolism
